# Sijunzi decoction granules for the treatment of advanced refractory colorectal cancer: study protocol for a multicenter, randomized, double-blind, placebo-controlled trial

**DOI:** 10.3389/fmed.2025.1523913

**Published:** 2025-03-20

**Authors:** Shuchang Nie, Yingyu Su, Lu Lu, Yanhua Jing, Zenghua Jiang, Yangxian Xu, Tingting Wu, Yi Zhong, Hao Wu, Junming Chen, Ming Ruan, Lan Zheng, Liyu Wang, Yabin Gong, Guang Ji, Hanchen Xu

**Affiliations:** ^1^Institute of Digestive Diseases, Longhua Hospital, China-Canada Center of Research for Digestive Diseases (ccCRDD), Shanghai University of Traditional Chinese Medicine, Shanghai, China; ^2^State Key Laboratory of Integration and Innovation of Classic Formula and Modern Chinese Medicine (Shanghai University of Traditional Chinese Medicine), Shanghai, China; ^3^Shanghai Frontiers Science Center of Disease and Syndrome Biology of Inflammatory Cancer Transformation, Shanghai, China; ^4^Department II of General Surgery, Longhua Hospital, Shanghai University of Traditional Chinese Medicine, Shanghai, China; ^5^Oncology Department, Shanghai Traditional Chinese Medicine - Integrated Hospital, Shanghai University of Traditional Chinese Medicine, Shanghai, China; ^6^Minhang Hospital, Fudan University, Shanghai, China; ^7^Department of Traditional Chinese Medicine, Ruijin Hospital, Shanghai Jiao Tong University School of Medicine, Shanghai, China; ^8^Oncology Department, Yueyang Hospital of Integrated Traditional Chinese and Western Medicine, Shanghai University of Traditional Chinese Medicine, Shanghai, China

**Keywords:** traditional Chinese medicine, Sijunzi decoction, randomized controlled trial, colorectal cancer, spleen deficiency syndrome

## Abstract

**Background:**

Colorectal cancer (CRC) ranks among the most common gastrointestinal cancers globally, with both its incidence and mortality rates showing an upward trend. In particular, the 5-year survival rate for stage IV CRC patients is only 14%. Conventional treatments such as chemotherapy and immunotherapy can lead to drug resistance, exacerbate gastrointestinal function damage, and induce immunosuppression. Sijunzi decoction (SJZD), as a fundamental formula of Traditional Chinese medicine (TCM), has been demonstrated to confer distinct advantages in treatment of CRC. Therefore, we designed this trial to explore the efficacy of SJZD for the treatment of advanced refractory CRC.

**Methods:**

A multicenter, randomized, double-blind, placebo-controlled trial is being conducted to assess the effectiveness of SJZD combined with standard therapy for treating advanced refractory CRC. Patients with advanced CRC will be recruited and randomly allocated to either the SJZD treatment group or the placebo group in a 1:1 ratio. Both groups will receive standard treatment. The intervention period will last for 6 months, with follow-up assessments every 8 to 10 weeks. Progression-free survival (PFS) is the main outcome measure. And the secondary outcomes contain duration of disease control (DDC), overall survival (OS), completion rate of chemotherapy, incidence of treatment-related adverse events, quality of survival scale score for tumor patients and changes in spleen deficiency patient-reported outcome (PRO) scores following the intervention.

**Expected outcomes:**

To the best of our knowledge, this trial marks the first clinical investigation into the therapeutic potential of SJZD for managing advanced refractory CRC. The primary aim of this study is to provide robust clinical evidence to support the integration of TCM with Western medicine in the treatment of advanced refractory CRC.

**Trial registration:**

The trial was registered at Chinese Clinical Trial Registry, http://www.chictr.org.cn (Registration No: ChiCTR2200065434); Date: 2022-11-04.

## Introduction

Colorectal cancer (CRC) is among the most common gastrointestinal malignancies worldwide (1, 2) and ranks third among the most common tumors in China, with increasing incidence and mortality rates (3, 4). Early stage CRC can be treated with radical surgery, with a 5-year survival rate of more than 90% (5). However, postoperative recurrence occurs in at least 40% of CRC patients (6, 7), and most patients are initially diagnosed at an advanced stage (8, 9), with unsatisfactory 5-year survival and overall mortality rates (10). In particular, the 5-year survival rate for patients with stage IV CRC is only 14% (11), and patients with RAS mutations have an even worse prognosis. Consequently, to identify effective treatment strategies for stage IV CRC patients with RAS mutations holds significant clinical importance.

Currently, for advanced CRC patients with RAS mutations, chemotherapy combined with targeted therapy has become the primary treatment. Nevertheless, standard medicine treatment has serious side effects, drug resistance and a poor prognosis. The long-standing history and proven efficacy of traditional Chinese medicine (TCM) make it a prominent treatment option for CRC in China. TCM has been demonstrated to confer distinct advantages in reducing toxicity, enhancing efficacy, preventing recurrence and metastasis, prolonging survival, and improving quality of life (12, 13).

Spleen deficiency syndrome (SDS) is recognized as one of the most common TCM syndromes in patients with CRC (14), and it mainly includes gastrointestinal symptoms such as anorexia and diarrhea, as well as systemic symptoms such as fatigue, perspiration, and aversion to cold. To enhance the accuracy of diagnosing this syndrome, a patient-reported outcome (PRO) scale specific to SDS has been developed. It includes 10 symptoms, each with its own weighting factor (Table 1) (15). A diagnosis of SDS is confirmed when the cumulative score of all symptoms exceeds 20. And our previous clinical study demonstrated the feasibility of this scoring scale (16).

**Table 1:** Spleen deficiency rating scale.

Questions	Scoring weight (%)
Do you feel tired and too lazy to speak?	20
Do you sweat easily?	5
Do you feel food is tasteless?	10
Are your stools thin and shapeless?	20
Do you drool a lot?	5
Do you have bleeding gums?	5
Do you feel your hands and feet are not warm?	10
Do you suffer from insomnia?	5
Do you feel you can easily catch a cold?	10
Do you have any abnormal changes in your eating habits recently, such as decreased appetite and abdominal distension after eating?	10

Sijunzi decoction (SJZD), consisting of four key ingredients—Radix Ginseng (Renshen), Rhizoma Atractylodis Macrocephalae (Baizhu), Poria (Fuling), and Radix Glycyrrhizae Preparata (Gancao)—is a foundational formula used in the clinical treatment of SDS. The standard dosages, as prescribed by classical texts and the 2015 Chinese Pharmacopoeia, are 9 g, 9 g, 9 g, and 6 g. Our preliminary clinical study showed that a Chinese herbal formula based on SJZD could extend the disease-free survival (DFS) of CRC patients after curative surgery. Chinese researchers have shown that SJZD can promote the apoptosis and autophagy of CRC cells through the PI3K/Akt/mTOR pathway, thus exerting a therapeutic effect on CRC (17). Several researchers have confirmed the effectiveness of SJZD in preventing and treating advanced CRC and studied its therapeutic targets and relevant mechanisms (17, 18). However, there are few clinical trials on SJZD for the treatment of CRC, so we designed a randomized, double-blind, placebo-controlled clinical trial to assess the efficacy and safety of SJZD-based integrated TCM combined with Western medicine for treating advanced refractory CRC.

## Methods and analysis

### Study design and setting

**Figure 1:**
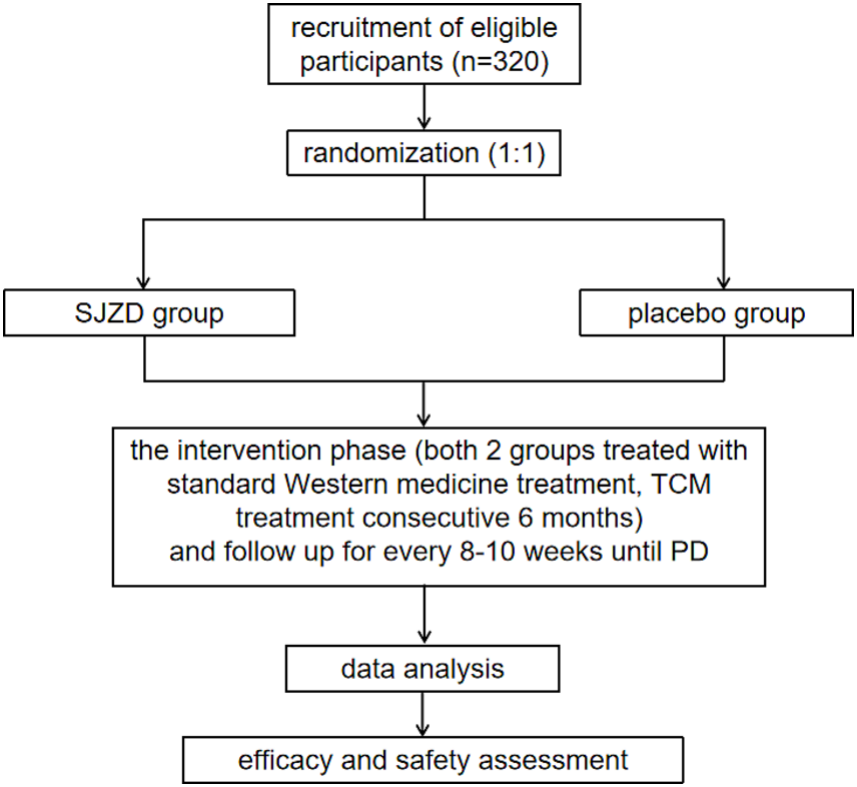
The flowchart of the trial.

This study protocol was approved by the Medical Ethics Committee of Longhua Hospital, affiliated with Shanghai University of Traditional Chinese Medicine (approval number: 2022LCSY082). And it was also registered with the Chinese Clinical Trial Registry (ChiCTR2200065434) on November 4, 2022.^1^ The trial flowchart is illustrated in Figure 1.

This is a multicenter, double-blind, randomized controlled trial involving two parallel groups. A total of 320 participants will be recruited through advertisements and referrals from clinicians in outpatient and inpatient settings. Patients who meet the inclusion criteria will be informed about the opportunity to participate in this study during their outpatient visits or upon admission. Detailed interviews will be conducted to explain the study’s objectives, interventions, examinations, and follow-up procedures. After obtaining informed consent, patients will be randomly allocated to either the SJZD group or the placebo group at a 1:1 ratio. During the intervention period, both groups will receive standard therapy. Additionally, the SJZD group will be administered SJZD granules, while the control group will receive a placebo as part of the treatment simulation. The key components of SJZD are outlined in Table 2. Treatment will continue for 6 months or until disease progression, resectable lesions are identified, patients are unable to tolerate the drug toxicity, patients withdraw their consent, patients are lost to follow-up, or patients die. Follow-up appointments will be scheduled every 8 to 10 weeks, as detailed in Table 3.

**Table 2:** Main components of Sijunzi decoction.

Chinese name	Latin name	Source plant	Amount (g)
Renshen	Ginseng Radix et Rhizoma	*Panax ginseng* C. A. Mey.	9
Baizhu	Atractylodis Macrocephalae Rhizoma	*Atractylodes macrocephala* Koidz.	9
Fuling	Poria	*Poria cocos(Schw.)*Wolf	9
Gancao	Glycyrrhizae Radix et Rhizoma	*Glycyrrhiza uralensis* Fisch.	6

**Table 3:** Schedule of trial.

Visit	Screening	Drug intervention period (weeks)			After drug withdrawal	Further follow-up (months)			
	0d	9 ± 1	18 ± 1	24 ± 1	6 month	9	12	15	18
Informed consent form	×								
Demographic information	×								
Inclusion and exclusion criteria	×								
General information	×								
Vital signs	×								
History of CRC and its treatment	×								
History of other diseases and their treatments	×								
Spleen deficiency rating scale	×	×	×	×		×	×	×	×
ECOG score	×	×	×	×		×	×	×	×
Routine blood tests	×	×	×	×		×	×	×	×
Routine urine test	×	×	×	×		×	×	×	×
Routine stool test	×	×	×	×		×	×	×	×
Liver function	×	×	×	×		×	×	×	×
Renal function	×	×	×	×		×	×	×	×
Coagulation function	×	×	×	×		×	×	×	×
Electrocardiograph	×	×	×	×		×	×	×	×
Quality of Survival Scale Score (EORTC QLQ-CR30)	×	×	×	×		×	×	×	×
Serum and stool samples collection	×				×				
Adverse events		×	×	×		×	×	×	×
Release drugs/placebo	×	×	×						
Retrieve empty packages		×	×	×	×				
Drug combination	×	×	×	×	×	×	×	×	×
Survival				×	×	×	×	×	×

### Objectives

The primary goal of this study is to assess the efficacy of integrating TCM with Western medicine, using the SJZD formula, in extending progression-free survival (PFS) and enhancing the quality of life for patients with advanced refractory CRC.

### Participants

Patients will be enrolled from oncology departments and Chinese Medicine departments in 5 hospitals—3 hospitals affiliated with the Shanghai University of Traditional Chinese Medicine, namely, Longhua Hospital, Yueyang Hospital of Integrated Traditional Chinese and Western Medicine, and Shanghai Traditional Chinese Medicine-Integrated Hospital; 1 hospital affiliated with the Shanghai Jiaotong University School of Medicine, Ruijin Hospital; and 1 hospital affiliated with Fudan University, Minhang Hospital.

### Inclusion criteria

Participants will be eligible if they meet all of the following inclusion criteria:

1. Patients with stage IV CRC and a definitive pathologic diagnosis of adenocarcinoma;
2. Presence of at least one clearly measurable tumor lesion as defined by RECIST 1.1 criteria;
3. Individuals aged over 18 years and of either sex;
4. Molecular pathology test results showing the RAS (including KRAS, NRAS and HRAS) mutant phenotype;
5. Organ function levels must meet the following requirements: (a) normal hematologic function: neutrophil count ≥1.5 × 10^9^/L, white blood cell count ≥3 × 10^9^/L, hemoglobin ≥90 g/L, and platelet count ≥75 × 109/L. (b) Hepatic function: total bilirubin ≤1.5 × upper limit of normal (ULN), ALT and AST ≤2.5 × ULN (≤5 × ULN for patients with liver metastases). (c) Renal function: serum creatinine ≤1.5 × ULN, and estimated creatinine clearance >50 mL/min. (d) Coagulation function: prothrombin time (PT) ≤1.5 × ULN, activated partial thromboplastin time (aPTT) ≤1.5 × ULN, and International Normalized Ratio (INR) ≤1.5 × ULN. (e) Electrolyte levels: serum magnesium and potassium ≥lower limit of normal (LLN); electrolyte correction is permitted during the screening period.
6. Patients who are proposed to receive first-line therapy or who have previously withdrawn from first-line therapy (regardless of molecularly targeted agent) due to intolerance of drug toxicity or imaging-confirmed disease progression or who have relapsed within 6 months of adjuvant chemotherapy completion;
7. Patients assessed as having spleen deficiency prior to enrolment for treatment;
8. ECOG performance status of 0 or 1;
9. Estimated life expectancy of at least 3 months;
10. Patients who have provided informed consent and are willing to participate in long-term follow-up.

### Exclusion criteria

Participants will be excluded if they meet any of the following criteria:

1. Histologic type of CRC other than adenocarcinoma;
2. Patients with wild-type RAS (including KRAS, HRAS and NRAS);
3. Bowel obstruction (except for patients whose obstruction has been relieved by fistula or stent placement) or active inflammatory bowel disease (patients who currently require medical intervention or are symptomatic) prior to study entry;
4. Pregnant women and lactating women;
5. Patients with mental disorders or other conditions that impede cooperation;
6. Active cardiovascular disease, such as cerebrovascular events, myocardial infarction, unstable angina, NYHA class II-IV congestive heart failure, or severe arrhythmias requiring medication that occurred within 6 months before the initiation of treatment in this study;
7. Uncontrollable high blood pressure;
8. Long-term use of high-dose aspirin (>325 mg/d);
9. Bleeding tendency or severe coagulation disorders;
10. Patients who cannot take medications orally, have structural abnormalities of the upper gastrointestinal tract, or suffer from malabsorption syndromes or other conditions that the investigators deem likely to interfere with gastrointestinal motility or absorption;
11. Patients with severe proteinuria (nephrotic syndrome);
12. Patients with a history of active tuberculosis (*Mycobacterium tuberculosis*) infection;
13. Patients with severe, nonhealing wounds, ulcers or fractures;
14. Patients with brain metastases, spinal cord compression, or primary brain tumors;
15. Patients with a history or physical evidence of uncontrolled central nervous system disorders (e.g., epilepsy or stroke);
16. Patients who have undergone surgery or open biopsy within 28 days prior to the initiation of study treatment;
17. Patients with a history of other malignancies within the past 5 years, excluding basal cell carcinoma of the skin and/or cervical carcinoma *in situ* following radical surgery;
18. Patients currently participating in other clinical trials;
19. Any other conditions where investigators determine the patient is unsuitable for participation.

### Withdrawal/termination criteria

Participants will be withdrawn or terminated from the trial if any of the following conditions are met:

1. Serious adverse events occur at any time, rendering patients unable to continue;
2. Patients decline to proceed with the clinical trial;
3. Patients who do not formally withdraw but discontinue medication and testing and are lost to follow-up.

Additionally, researchers will document the last date of drug administration and collect assessment data for participants who discontinue or deviate from the intervention protocols.

### Interventions

#### Drug intervention

**TCM intervention**: The intervention phase will span 6 months. Both the SJZD and placebo are formulated as granules, each weighing 1.77 g per pack and packaged identically to ensure uniform appearance, texture, smell, and taste. However, the placebo granules lack therapeutic effects. Patients will be instructed to dissolve each pack of granules in 200 mL of hot water and take two packs orally twice daily (totaling 3.54 g/day). The dosage and frequency will remain constant throughout the study. The SJZD and placebo granules are supplied by Sichuan Neo-green Pharmaceutical Technology Development Co., Ltd. (Sichuan, China).

The ingredients of the placebo are a one-tenth dose of SJZD (Radix ginseng, Rhizoma Atractylodis macrocephalae, Poria and Radix Glycyrrhizae Preparata) and maltodextrin. All of the processing and preparation of the medications and placebo will be conducted by qualified personnel.

Participants experiencing severe adverse events (AEs) related to this trial will receive posttrial care along with additional compensation if necessary. For participants who have adverse reactions unrelated to the trial medication, the relevant medical interventions will be implemented based on the comprehensive information of the patients, including their previous medical history, symptoms and signs, and laboratory, imaging and other examination results.

The placebo composition includes a one-tenth dose of SJZD along with maltodextrin. All processing and preparation of both the medications and placebo will be conducted by qualified personnel.

Participants who experience severe adverse events (AEs) related to this trial will receive post-trial care and additional compensation if necessary. For participants with adverse reactions not associated with the trial medication, appropriate medical interventions will be implemented based on comprehensive patient information, including their medical history, symptoms, signs, and results from laboratory tests, imaging, and other examinations.

**Standard medicine intervention**: The standard treatment will follow the recommendations outlined in the National Comprehensive Cancer Network (NCCN) Guidelines (2022 edition).

1. **First-line chemotherapy regimen:**
2. FOLFOX+ Bevacizumab [oxaliplatin 85 mg/m^2^, ivgtt for 2 h, Day 1; calcium folinate 400 mg/m^2^, ivgtt for 2 h, Day 1; fluorouracil 400 mg/m^2^, i.v., Day 1, 1,200 mg/(m^2^d) × 2 d, continuous ivgtt]; (total 2,400 mg/m^2^ for 46–48 h); bevacizumab 5 mg/kg, ivgtt, Day 1; a course of 14 days, with an expected total of 12 chemotherapy treatments;
3. FOLFIRI+ Bevacizumab [irinotecan 180 mg/m^2^, ivgtt for 30–90 min, Day 1; calcium folinate 400 mg/m^2^, ivgtt for 2 h, Day 1; fluorouracil 400 mg/m^2^, i.v., Day 1, 1,200 mg/(m^2^d) × 2 d, continuous ivgtt]; (total 2,400 mg/m^2^ for 46–48 h); bevacizumab 5 mg/kg, ivgtt, Day 1; a course of 14 days, with an expected total of 12 chemotherapy treatments;
4. CAPEOX+ Bevacizumab (oxaliplatin 130 mg/m^2^, ivgtt for more than 2 h, Day 1); capecitabine was taken orally 1,000 mg/m^2^ twice a day (once in the morning and once after meals) for the 1st to 14th days; bevacizumab 5 mg/kg, ivgtt, Day 1; a course of 21 days, with an expected total of 8 chemotherapy treatments.
5. **Second-line chemotherapy regimen:**
6. First-line treatment with oxaliplatin: FOLFIRI+ Bevacizumab;
7. First-line treatment with irinotecan: FOLFOX/CAPEOX+ Bevacizumab;
8. First-line treatment without irinotecan or oxaliplatin: FOLFOX/FOLFIRI/CAPEOX+ Bevacizumab.
9. **Maintenance therapy:** Maintenance therapy is given to patients whose disease has not progressed and who tolerate the drug well after the completion of standard chemotherapy. Maintenance therapy is continued until the disease has progressed, the lesion is resectable, the patient is unable to tolerate the toxic effects of the drug, or the patient withdraws consent, is lost to follow-up, or dies.

Maintenance treatment options:

1. FOLFOX+ Bevacizumab [calcium folinate 400 mg/m^2^, ivgtt for 2 h, Day 1; fluorouracil 400 mg/m^2^, i.v., Day 1, 1,200 mg/(m^2^d) × 2 d, continuous ivgtt]; (total 2,400 mg/m^2^ for 46–48 h); bevacizumab 5 mg/kg, ivgtt, Day 1; 14 days for each course of treatment;
2. FOLFOX+ Bevacizumab [calcium folinate 400 mg/m^2^, ivgtt for 2 h, Day 1; fluorouracil 400 mg/m^2^, i.v., Day 1, 1,200 mg/(m^2^d) × 2 d, continuous ivgtt]; (total 2,400 mg/m^2^ for 46–48 h); bevacizumab 5 mg/kg, ivgtt, Day 1; 14 days for each course of treatment;
3. CAPEOX+ Bevacizumab (capecitabine taken orally 1,000 mg/m^2^ twice a day; once in the morning and once after meals) for the 1st to 14th days; bevacizumab 7.5 mg/kg, ivgtt, Day 1; 21 days for each course of treatment.

### Concomitant treatment regulations

During the trial, participants will be prohibited from using any other medications or interventions that could influence the evaluation of efficacy and safety. For participants with coexisting chronic conditions requiring ongoing treatment, any concurrent interventions must be documented in the case report form (CRF).

## Outcomes

The primary outcome measure is PFS. This is defined as the time interval from the date of patient enrollment to the first occurrence of malignant tumor progression or death from any cause.

The secondary outcomes include the following: (1) duration of disease control (DDC); (2) overall survival (OS); (3) completion rate of chemotherapy; (4) incidence of treatment-related adverse events; (5) quality of survival scale score for tumor patients, European Organization for Research and Treatment of Cancer Quality of Life Questionnaire-Colorectal 30 (EORTC QLQ-CR30); and (6) changes in the spleen deficiency PRO scores following the intervention.

The safety assessment will include physical examinations, monitoring of vital signs, and laboratory tests at each visit, along with ongoing surveillance of AEs during the intervention period. The laboratory assessments will include routine blood, urine, and stool analyses; evaluations of liver and renal function; and electrocardiograms (ECGs). AEs will be defined and graded in accordance with the National Cancer Institute’s Common Terminology Criteria for Adverse Events (CTCAE), Version 5.0 (US Department of Health and Human Services, 2017).

### Randomization and blinding

To ensure balanced allocation, a permuted block randomization method, stratified by the recruitment locations, will be employed to assign participants to either the SJZD group or the placebo group in a 1:1 ratio using the SAS statistical software. Each medication dose will be placed in identical opaque envelopes marked with distinct randomized identification numbers. Participants will be enrolled sequentially by clinical staff and assigned an envelope based on their order of entry into the study. An independent statistician will manage the randomization procedure. All individuals involved in the trial—including clinicians, nurses, researchers, and patients—will remain unaware of the treatment assignments. The randomization codes and treatment allocations will be securely stored by the principal investigators until the trial and data analysis are completed, except in cases of serious AEs. In the event of such occurrences, sealed emergency letters containing unblinding information will be distributed to each recruitment site as needed. These letters will provide details about the treatment assignment and any urgent conditions. If any serious AEs arise, the emergency letter will be opened, and the affected participant will be withdrawn from the study. A report of any serious AEs must be submitted to the principal investigators within 24 h. After the follow-up period ends, the treatment codes will be disclosed, and the specific interventions associated with these codes will be revealed following the completion of data analysis.

### Sample size

The sample size for this study was calculated based on the primary efficacy indicators. According to previous cohort studies conducted by the research team of Longhua Hospital, it was found that patients with advanced CRC who received a combination of traditional Chinese medicine and standard Western medicine had a median PFS of 9.21 months. In contrast, the median PFS for patients treated solely with standard Western medicine was 7.5 months. Following the 1:1 parallel control principle, SAS statistical analysis software was used to generate the sample size with a double-sided significance level of 0.05 and a power of 80% (*α* = 0.05, *β* = 0.2). Assuming that the dropout rate in each group will be 10%, 320 patients are required (*n* = 160 in each group). In accordance with the 1:1 parallel control design, we utilized SAS statistical software to calculate the required sample size, considering a two-sided significance level of 0.05 and a power of 80% (*α* = 0.05, *β* = 0.2). Anticipating a dropout rate of approximately 10% in each group, the total number of patients needed is 320, with 160 patients in each arm of the study.

### Data collection and registration

Information will be documented in CRF and subsequently uploaded to REDcap,^2^ a web-based electronic data management system. All clinical investigators will receive training on patient communication, information collection, and inputting data into the electronic database. Researchers will secure informed consent from participants, evaluate their general and demographic details, and gather baseline biomedical and tumor-related data.

Individuals meeting the NAE criteria will be recruited and started chemotherapy in combination with the herbal/placebo treatment. After enrolment and initiation of treatment, participants will undergo follow-up assessments every 8 to 10 weeks, and the treatment will be continued until the disease progresses, the lesions are resectable, the patients cannot tolerate the toxic side effects of drugs, or the patients withdraw their consent, are lost to follow-up, or die. During the course of the study, if the patient’s CEA level continues to increase abnormally or if other evidence of a suspected recurrence is found, the frequency of follow-up will be accelerated promptly. Additionally, physical examination, colonoscopy, chest/abdominal/pelvic CT scanning with enhancement, and PET-CT will be performed if necessary.

The initial data and collected samples will be retained for a minimum of 5 years following the completion of the trial. An independent Data Monitoring Committee (DMC) will be formed, consisting of clinical epidemiologists, data monitors, and statisticians who are not involved in the trial and have no affiliation with the sponsors. This committee will ensure there are no conflicts of interest. The DMC will conduct biannual reviews of documents, CRFs, and relevant data to oversee trial safety, verify the accuracy of participant profiles, and safeguard the confidentiality of participants’ information. Additionally, the DMC will offer recommendations regarding potential modifications to the study design or considerations for trial termination.

### Statistical analysis

Data analysis will be conducted using SAS statistical software. The efficacy and safety of the study will be evaluated according to the intention-to-treat (ITT) principle. Missing data will be imputed using the last-observation-carried-forward method. Continuous variables will be presented as means ± standard deviations or medians (interquartile ranges), depending on the distribution and uniformity of variance. Categorical variables will be compared between the SJZD and placebo groups using the χ^2^ test. Within-group comparisons will be analyzed using paired t-tests, while between-group comparisons will employ independent sample t-tests. Logistic regression analysis will be utilized to assess factors influencing the recurrence rate. An interim analysis is planned after 50% of the sample has been collected. Subgroup analyses will be conducted based on participant characteristics such as sex, chemotherapy regimen, and tumor stage. A *p*-value of less than 0.05 will be considered statistically significant.

### Trial status

Participant enrollment is scheduled to begin in January 2023 and conclude by March 2025. Follow-up assessments are expected to be finalized by December 2025. Clinical data will be secured and locked in January 2026. This study protocol was submitted prior to the completion of participant recruitment.

### Ethics and dissemination

This research adheres to the guidelines outlined in the Declaration of Helsinki, the Standard Protocol Items: Recommendations for Interventional Trials (SPIRIT) 2013 Statement, and the Standard Protocol Items for Clinical Trials with Traditional Chinese Medicine: Recommendations, Explanation and Elaboration (SPIRIT-TCM) Extension 2018 Statement. The study protocol received approval from the ethics committee of Longhua Hospital, affiliated with Shanghai University of Traditional Chinese Medicine (Approval No. 2022LCSY082), and is registered with the Chinese Clinical Trial Registry (ChiCTR2200065434). Any modifications to the study design will be promptly reported to the ethics committee. Enrollment will be restricted to individuals who have provided written informed consent.

Upon completion of the study, the findings will be submitted for publication in a peer-reviewed journal. Clinical insights and trial experiences will be shared with participants and the broader public via conferences and publications. Authorship of the final report will be determined based on individual contributions to the study.

## Discussion

The incidence of CRC remains a significant global health challenge, with over 1.9 million cases reported worldwide in 2020, resulting in approximately 935,000 deaths, accounting for approximately one-tenth of all cancer cases and deaths (19). Furthermore, the burden of CRC is projected to increase to 3.2 million new cases and 1.6 million deaths by 2040 (20). Consequently, the management of CRC presents an urgent clinical challenge. Current standard therapies for CRC encompass a range of approaches, including surgical intervention, chemotherapy, immunotherapy, targeted therapy, radiation therapy, and various combinations thereof.

Surgery is the primary treatment for CRC; however, serious complications, such as anastomotic leakage, intestinal obstruction, and hernia, are common after surgery (21) and can lead to gastrointestinal dysfunction and abnormal nutrient metabolism.

Recent advances in tumor immunotherapy have been remarkable. Pembrolizumab improves the median survival time of patients with metastatic CRC from 8.2 to 16.5 months and has become the standard first-line treatment for microsatellite instability-high (MSI-H) and mismatch repair-deficient (dMMR) metastatic CRC (22). However, immune checkpoint inhibitors (ICIs) are only effective in CRC patients with MSI-H/dMMR, which accounts for only about 15% of CRC cases (23). Targeted therapies such as cetuximab and bevacizumab have significantly improved OS for patients, but resistance can easily develop (24).

In recent years, scientists have studied novel molecular factors (KRASs, BRAFs, and microsatellite instability markers) to improve the prognostic stratification and personalized treatment of CRC (25). KRAS mutations are found in approximately 40% of CRC patients (all stages) and are associated with a poor prognosis. According to the National Comprehensive Cancer Network (NCCN) guidelines, only patients with wild-type RAS should be treated with anti-EGFR inhibitors after evaluation of RAS mutations, as BRAF and RAS mutations (including mutations in KRAS and NRAS) are associated with primary resistance to EGFR therapy (26, 27). Unfortunately, until recently, no drugs were available to directly target mutant KRAS (28). Currently, the most effective treatment for advanced CRC is based on the combination of fluorouracil and oxaliplatin (FOLFOX/XELOX) or fluorouracil and irinotecan (FOLFIRI) (29, 30). With the development of targeted drugs such as bevacizumab and cetuximab, a standardized treatment scheme for advanced CRC involving the combination of chemotherapy with targeted therapy has been developed (31). However, chemotherapy can cause drug resistance, worsen gastrointestinal function damage, and cause immunosuppression (32, 33), leading to a series of adverse reactions in elderly or weak patients and preventing them from tolerating further chemotherapy, ultimately resulting in a poor prognosis (34).

TCM is an important component of comprehensive treatment for cancer, and it has accumulated rich experience in the long-term treatment of malignancies (35, 36). TCM has unique advantages in reducing toxicity, enhancing efficacy, preventing recurrence and metastasis, prolonging survival, improving the quality of life for patients treated with chemotherapy, reducing adverse reactions, and enhancing immunity (12, 13). Patients with advanced CRC are mostly in the pathological stage of deficiency of positive qi and residual evils, which is in line with the pathological mechanism and clinical manifestations of SDS. Treatment is mainly based on supporting positive qi, and the nutritional status and immune function of patients can be improved through tonic formulae. SDS is recognized as one of the most common TCM syndromes in patients with CRC (14). Additionally, a study suggested that patients with advanced CRC had higher SDS scores than patients with early-stage CRC (37).

SJZD, a classical prescription for treating SDS in China for centuries, has been widely used for gastrointestinal diseases and is favored by clinicians due to its adaptability with the addition of other Chinese herbs under different pathological conditions (38). Wu and Xuan reported that SJZD could enhance the clinical symptoms of CRC patients, improve their quality of life and survival rate, and decrease the risk of tumor recurrence and metastasis (39). Shang demonstrated that SJZD induces autophagy and apoptosis in HCT116 and LOVO cancer cells by modulating PI3K/Akt/mTOR signaling in CRC (17). Another study confirmed that the core genes HSPB1, SPP1, IGFBP3 and TGFB1, which are involved in the therapeutic mechanism of SJZD in the CRC TME, affect CRC development and prognosis by regulating hypoxia, protein binding and EMT in the extracellular matrix (40). Modified SJZ was observed to have a suppressive impact on liver metastasis of colon cancer *in vivo*, both as a standalone treatment and in combination with 5-Fu. The potential mechanism may be attributed to its ability to stimulate cytokines such as GM-CSF, leading to an increase in the number of macrophages in the spleen and the timely clearance of colon cancer cells from the vascular system (41).

Nevertheless, randomized clinical trials evaluating the combination of TCM with standard therapies for advanced CRC remain limited. Consequently, we designed this study to investigate the efficacy and safety of SJZD in treating patients with refractory advanced CRC.

This study has several limitations. Firstly, the design does not include planned dose reduction or escalation, precluding a comparison of dose–response effects. Secondly, all participating sites are located in Shanghai, which limits the generalizability of the findings. Future studies should aim for a larger and more diverse sample size to enhance external validity.

In conclusion, this trial will employ a randomized, double-blind, placebo-controlled design to investigate a combination of TCM and Western medicines for the treatment of advanced refractory CRC, using SJZD as the foundational formula. The study aims to assess the efficacy and safety of this combined approach in managing advanced CRC, with PFS serving as the primary efficacy endpoint, aiming to provide a robust scientific foundation for the clinical application of TCM and Western medicine in the diagnosis and treatment of CRC.

## Footnotes

^1^ http://www.chictr.org.cn/listbycreater.aspx

^2^ http://longhua.site/redcap3
